# Microscale thermophoresis as a powerful tool for screening glycosyltransferases involved in cell wall biosynthesis

**DOI:** 10.1186/s13007-020-00641-1

**Published:** 2020-07-28

**Authors:** Wanchen Shao, Rita Sharma, Mads H. Clausen, Henrik V. Scheller

**Affiliations:** 1 Joint BioEnergy Institute, Emeryville, CA 94608 USA; 2 Environmental Genomics and Systems Biology Division, Lawrence Berkeley National Laboratory, Berkeley, CA 94720 USA; 3 School of Computational & Integrative Sciences, Jawaharlal Nehru University, New Delhi, 110067 India; 4 Department of Chemistry, Technical University of Denmark, 2800 Kgs. Lyngby, Denmark; 5 Department of Plant and Microbial Biology, University of California, Berkeley, CA 94720 USA

**Keywords:** Plant cell wall, Glycosyltransferase, *At*GALS1, Microscale thermophoresis, Sorghum, GT61

## Abstract

**Background:**

Identification and characterization of key enzymes associated with cell wall biosynthesis and modification is fundamental to gain insights into cell wall dynamics. However, it is a challenge that activity assays of glycosyltransferases are very low throughput and acceptor substrates are generally not available.

**Results:**

We optimized and validated microscale thermophoresis (MST) to achieve high throughput screening for glycosyltransferase substrates. MST is a powerful method for the quantitative analysis of protein–ligand interactions with low sample consumption. The technique is based on the motion of molecules along local temperature gradients, measured by fluorescence changes. We expressed glycosyltransferases as YFP-fusion proteins in tobacco and optimized the MST method to allow the determination of substrate binding affinity without purification of the target protein from the cell lysate. The application of this MST method to the β-1,4-galactosyltransferase *At*GALS1 validated the capability to screen both nucleotide-sugar donor substrates and acceptor substrates. We also expanded the application to members of glycosyltransferase family GT61 in sorghum for substrate screening and function prediction.

**Conclusions:**

This method is rapid and sensitive to allow determination of both donor and acceptor substrates of glycosyltransferases. MST enables high throughput screening of glycosyltransferases for likely substrates, which will narrow down their in vivo function and help to select candidates for further studies. Additionally, this method gives insight into biochemical mechanism of glycosyltransferase function.

## Background

Glycosylation is a fundamental process in biology, conserved across all domains of life. Proteins, lipids, and small molecules are frequently glycosylated, and polysaccharides are ubiquitous to all organisms [1]. The principal enzymes responsible for glycosylation are glycosyltransferases (GTs). GTs use activated sugars, usually nucleotide sugars (NDP-sugars), as donor substrate [2]. There are about 10–15 common NDP-sugars used by GTs whereas potential acceptor substrates are unlimited [3]. While GTs can often be recognized by their primary structures their specificity cannot currently be predicted, and elucidating GT function has been excruciatingly slow. In plants less than 10% have a known function [4]. Unique assays must be developed for every glycosylated product studied. Determining the function of a GT currently requires correct identification of both donor and acceptor substrates in addition to the development of an assay for product identification. Some commercial (UDP/CMP/GDP-Glo™, Promega) and reported glycosylation assays are based on detection of UDP or GDP released during the reaction through coupling to another reaction [5, 6] making them more suitable for high throughput studies, but they still depend on acceptor substrates. Often acceptor substrates are not only unknown, but candidate substrates are generally not available from commercial and other sources.

A large proportion of the GTs in plants are known or likely to be involved in cell wall biosynthesis. The plant cell wall, an extracellular matrix of polysaccharides and glycoproteins, is crucial for plant growth, morphology, integrity, and biomass recalcitrance [7, 8]. Dynamic structural change in the plant cell wall promotes plant development through deposition, crosslinking, remodeling, and degradation [9, 10]. Polysaccharides of the plant cell wall comprises cellulose synthesized at the plasma membrane and matrix polysaccharides, which are synthesized in the Golgi apparatus and transported by endo-membrane vesicles within the secretory pathway for deposition [11]. During this process, biosynthetic GTs are the main enzymes required for the synthesis of the polysaccharides. In order to gain insights into cell wall biology, it is fundamental to identify and characterize key GTs associated with cell wall biosynthesis and modification.

However, long-standing challenges to characterizing these Golgi-localized GTs exist. First, protein purification for biochemical activity assay has only been accomplished in few cases due to the labile nature and low abundance of these enzymes [12, 13]. Heterologous expression in *E. coli* has been largely ineffective, likely limited by insufficient folding and post-translational modification in bacterial systems [14–16]. Heterologous expression in eukaryotic systems has been reported, but no generally successful system has been found. Second, activity assays are often difficult since assays must use both nucleotide-sugar donor and acceptor substrates, which are generally not available. Finally, based on sequence homology, GTs have been classified into 110 families in the Carbohydrate Active enZyme database (CAZy, http://www.cazy.org) [17]. For each GT family at least one member was characterized biochemically, whereas most of the other family members have been assigned based on their sequence. Substrate specificity, however, is very difficult to predict from the sequence. Many GT families contain enzymes with widely different activities despite the sequence similarity. When there is no clear hypothesis for the function of a GT it is essentially impossible to determine both the sugar donor and acceptor. Even when mutant studies or sequence similarity lead to hypothesis of potential activity, it can be difficult to know if inability to detect activity is due to the wrong substrates being tested or a problem with the activity of the heterologously expressed enzyme. Indeed, some members of GT families may not have GT activity in vivo but could have other roles, e.g. as part of GT complexes [18, 19]. As a result, there are significantly fewer well characterized GTs compared to the large number predicted by bioinformatic methods. However, the nucleotide sugar donor substrates constitute a limited set of about thirteen common ones in plants (UDP-α-d-Glc, UDP-α-d-Xyl, UDP-β-l-Ara_*f*_, UDP-β-l-Ara_*p*_, UDP-α-d-Gal, UDP-β-l-Rha, UDP-α-d-GlcNAc, GDP-α-l-Gal, GDP-β-l-Fuc, GDP-α-d-Man, GDP-α-d-Glc, UDP-α-d-GalA, UDP-α-d-GlcA). Less common nucleotide sugars in plants include CMP-β-d-Kdo, UDP-α-d-Api and the unknown precursors of Dha (2-keto-3-deoxy-d-lyxo-heptulosaric acid) and Ace (3-C-carboxy-5-deoxy-l-xylose). Recent evidence indicates the presence of UDP-α-d-Fuc at least in some plants [20], but d-Fuc is not known to be part of any cell wall polysaccharide. A method to screen for putative donor substrate in the absence of acceptor would therefore be extremely useful in initial testing of putative biochemical function and in validating that a heterologously expressed enzyme is likely to be active. Acceptor substrates are much more diverse, but a method to screen putative acceptor substrates in the absence of donor would likewise be extremely useful.

**Fig. 1:**
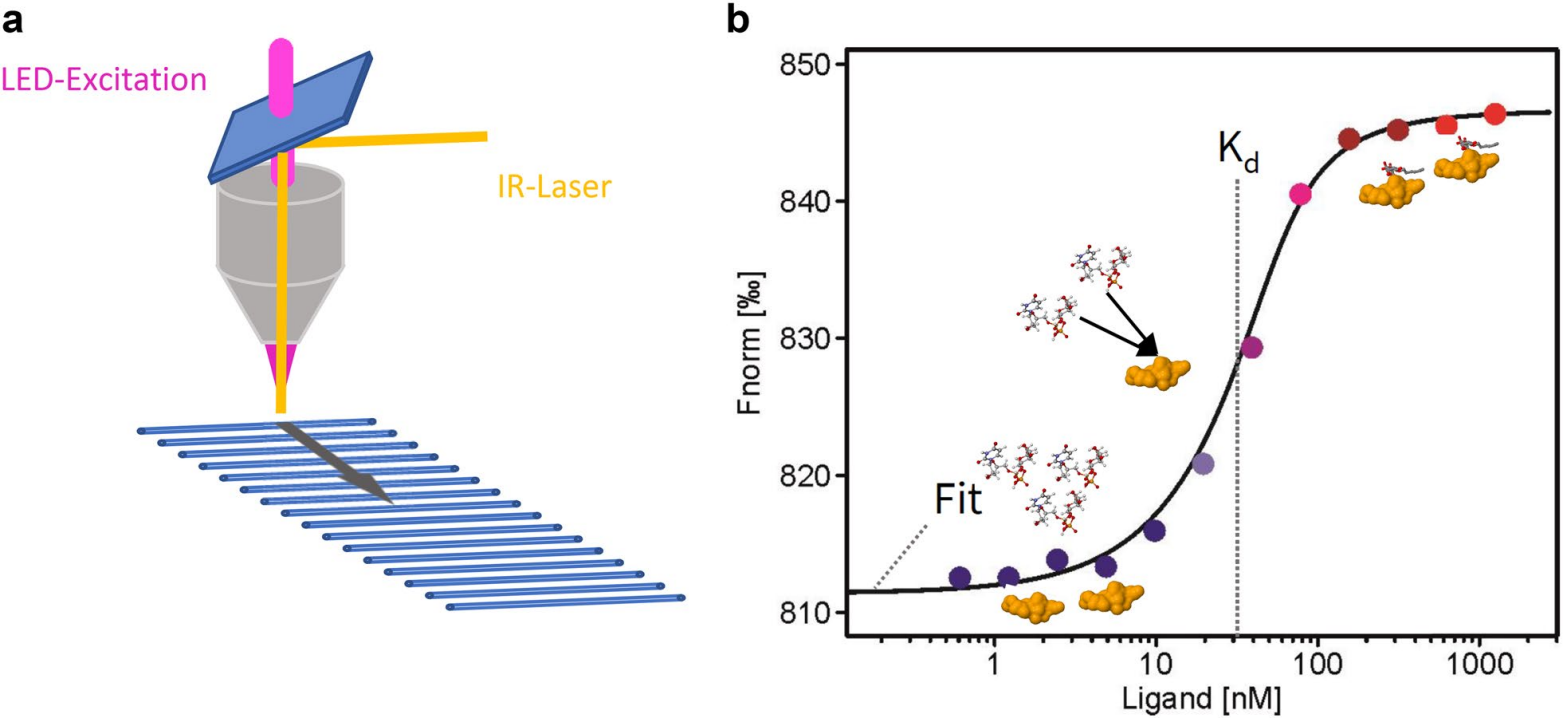
Setup of the apparatus to determine the thermophoresis of biomolecules. **a** An infrared (IR) laser heats the aqueous sample filled in a thin glass capillary and generates a localized microscopic temperature gradient in the range of 2–6 °C. Protein complexes with interaction partners demonstrate slower movement through the temperature gradient compared with free molecules. This movement is monitored via fluorescence of the target protein. Rapid scanning of 16 capillaries loaded with fluorescent target protein at a constant concentration and substrates in increasing concentration gradients enables the determination of equilibrium binding constants. **b** A binding curve can be calculated from the gradual difference of thermophoresis between the fluorescent molecules of both unbound and bound states, which is plotted as Fnorm, defined as F_hot_/F_cold_ against the ligand concentration. The binding constant K_d_ can be then derived from the binding curve

An emerging and sensitive method for studying molecular interactions is microscale thermophoresis (MST). The technique is based on the motion of molecules along local temperature gradients, measured by fluorescence changes [21, 22]. A spatial temperature difference (Δ*T)* leads to a mass flow of molecules in the temperature-elevated region. The Soret coefficient, also known as thermophoresis, was defined by *S*T: *c*_hot_ = *c*_cold_ * exp(− *S*T  Δ*T* ) and quantified the relation of temperature gradient and the molecule flow [23, 24]. The thermophoresis of a protein typically differs significantly from the thermophoresis of a protein–ligand complex due to binding-induced changes in size, charge, and hydration shell [24]. Figure 1 shows the setup of the instrument for MST.

With no size limitations of the interacting molecules, this technique makes it possible to study interactions between GTs and their substrates without requiring much sample volume. Here we optimized and validated MST to facilitate high throughput screening for GT substrates. We expressed GTs as YFP-fusion proteins in tobacco (*Nicotiana benthamiana*) and optimized the MST method to allow the determination of substrate binding affinity, without purification of the target protein from the cell lysate. The application of this MST method to the β-1,4-galactosyltransferase *At*GALS1 validated the capability to screen nucleotide-sugar substrates as well as acceptor substrates. We also expanded the application to the identification of GTs in sorghum, which will enable selection of candidates for further studies and engineering.

## Results

### MST assay optimization of reaction buffer formulation

To determine the best buffer formulation for analyzing GT-substrate interaction, several buffers and non-ionic detergents were pre-tested based on the produced florescence counts and thermograph profiles during thermophoresis. The well-characterized *Arabidopsis thaliana* β‐1,4‐galactan synthase 1 (*At*GALS1), which catalyzes the elongation of pectic β‐1,4‐galactan chains using UDP-Gal as donor substrate [25], was used to test the buffer formulation and conditions. While N-terminal tags may in some cases interfere with targeting, YFP-*At*GALS1 is known to be efficiently targeted to Golgi and we therefore used this well-characterized construct [25]. Since divalent cations are commonly required for GT function, and are known to be essential for *At*GALS1 activity [26], all the buffers tested contained 5 mM Mn^2+^. Microsomes extracted from *N. benthamiana* leaves transiently expressing YFP-*At*GALS1 were used as a source of *At*GALS1 protein. Extracted microsomes were diluted to reach an optimal fluorescence level between 200 and 1600 units of fluorescence (F1 units). The final concentration of the total microsomal protein was kept constant at 1.5 mg/ml. The concentration of *At*GALS1 is not known, but assuming that it may constitute 1% of total microsomal protein, the final concentration would be in the order of 250 nM. As shown for YFP-*At*GALS1, MES or HEPES buffer with 1% Triton X100 provided the highest fluorescent counts (Table 1), allowing further dilution of the *At*GALS1-containing microsomes if necessary. The MES buffer also presented a smooth curve with no indication of protein aggregation that can hinder the interpretation of measurements (Table 1, Additional file 1: Figure S1). A buffer with Tween20 with concentrations ranging from 0.05 to 2% all yielded much lower fluorescence counts and signs of aggregation compared to that using a Triton X100-based buffer, suggesting Triton X100 serves as better detergent in this case (Table 1, Additional file 1: Figure S1). Since aggregation can also be caused by too low of a salt concentration and resulting charge-to-charge interaction of biomolecules, adding 125 mM KCl to the MES buffer was also tested. However, this also resulted in thermograph profile suggesting protein aggregation (Table 1, Additional file 1: Figure S1).

**Table 1:** Fluorescence counts and aggregation status of YFP-AtGALS1 microsomes in different incubation buffers

Buffer composition	Fluorescence counts	Aggregation observed
HEPES pH 7.0, 50 mM NaCl, 5 mM MnCl_2_, 1% Tx100	1247	(+)
PBS pH 7.4, 5 mM MnCl_2_, 0.05% Tween	479	−
MES pH 6.5, 5 mM MnCl_2_, 125 mM KCl, 1% Tx100	974	+
MES pH 6.5, 5 mM MnCl_2_, 1% Tx100	1263	−
MES pH 6.5, 5 mM MnCl_2_, 0.05% Tween	101	++
MES pH 6.5, 5 mM MnCl_2_, 0.5% Tween	174	+
MES pH 6.5, 5 mM MnCl_2_, 2% Tween	272	−

MES buffer with 1% Triton X-100 provides highest fluorescence counts and did not produce bumpy thermograph curve as indication of protein aggregation. Tween as detergent with concentration from 0.05 to 2% did not work well with MES. Thermographs corresponding to the different conditions are shown in Additional file 1: Figure S1

### Method validation with YFP-*At*GALS1 binding its substrate UDP-Gal

**Fig. 2:**
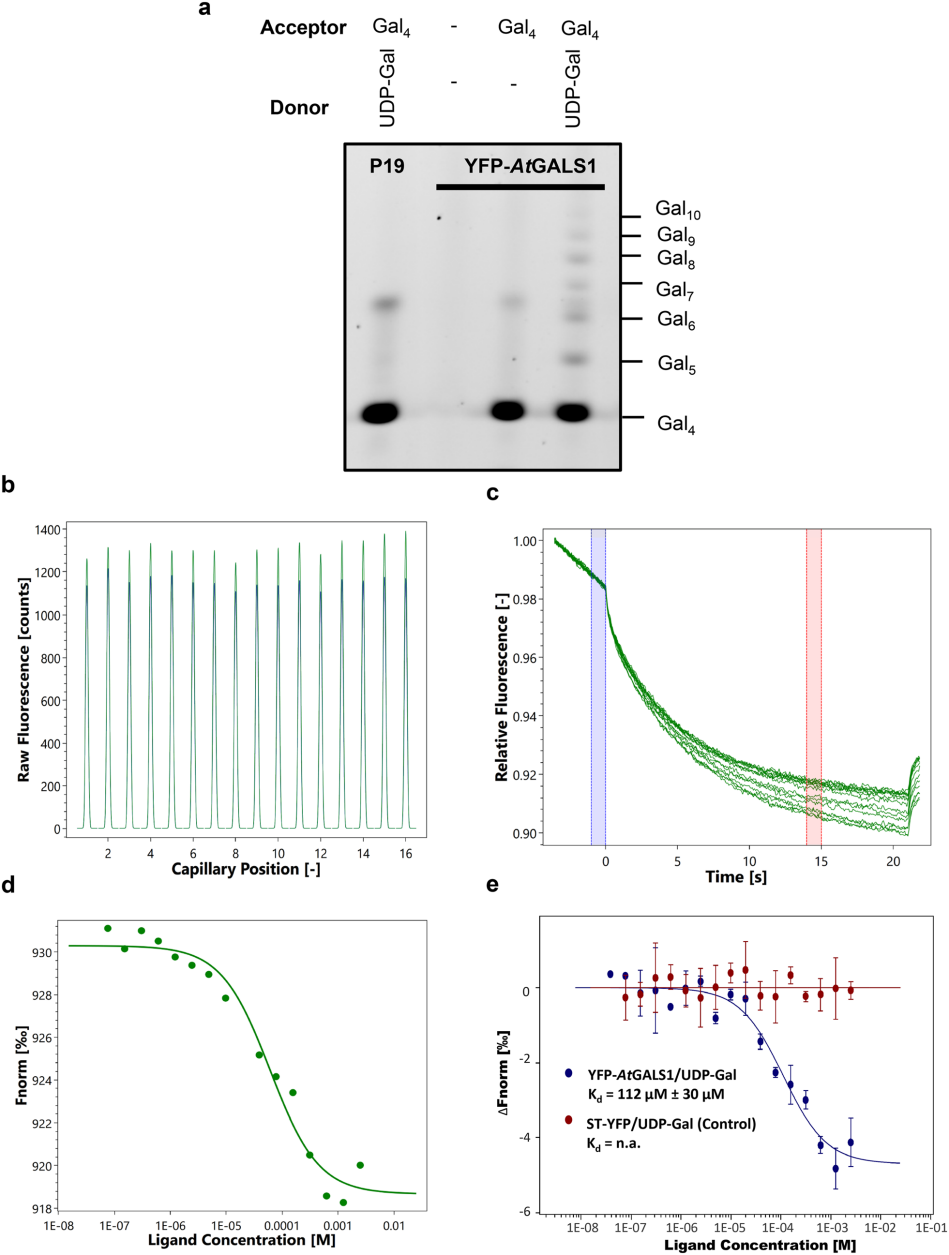
Substrate binding assay of *At*GALS1. **a** Activity analysis of *At*GASL1 using carbohydrate gel electrophoresis (PACE). Incubation of YFP-*At*GALS1 microsomes and ANTS-labeled β-1,4-galactotetraose (Gal_4_) in the presence and absence of 200 μM UDP-Gal displayed activity of catalyzing the elongation of galactan backbone. No activity was observed in the p19 control. **b** Initial fluorescence counts of YFP-*At*GALS1 microsomes in MES buffer at different concentrations of UDP-Gal. The variation in fluorescence across the concentration gradients is within the tolerance range (± 10%). **c** Thermographs of YFP-*At*GALS1 binding to UDP-Gal provide well-defined curves. The blue region at 0 s indicates cold spot before the temperature gradient was applied, and the red region at 15 s shows the hot spot during the thermophoresis. **d** Dose–response curve for the binding interaction between YFP-*At*GALS1 and UDP-Gal by plotting Fnorm against the ligand concentration. The binding curve yields a K_d_ of 101 μM. **e** Normalized binding curve of YFP-*At*GALS1 and ST-YFP control in presence of UDP-Gal. The binding curve yields a K_d_ of 112 ± 30 μM. Concentration of YFP-*At*GALS1 or ST-YFP were kept constant while the UDP-Gal concentration varied from 2.5 mM to 76.3 nM. Data were fitted to K_d_ model and confidence interval of the K_d_ is calculated from the variance of the fitted parameter, derived from Levenberg–Marquardt fit-algorithm. Error Bar: SD, n = 3. Values are the average ± 68% confidence

To analyze GT-substrate interaction using MST, *At*GALS1 was tested for binding to its substrate UDP-Gal. To first validate that YFP-*At*GALS1-containing microsomes retained the native protein confirmation and activity of *At*GALS1, we employed Polysaccharide Analysis using Carbohydrate gel Electrophoresis (PACE) [27] to detect the enzymatic activity. A PACE assay using Gal_4_ substrate conjugated with the fluorophore ANTS (8-amino-naphthalene-1,3,6-trisulfonic acid) [28] in the presence of 200 μM UDP‐Gal as donor sugar displayed elongation activity, and produced a galactan chain with maximum detected degree of polymerization (DP) of approximately 11 (Fig. 2a). A control with microsomes from leaves expressing p19 alone revealed no activity.

With an activity-validated microsome preparation, we proceeded to use MST to detect the interaction of *At*GALS1 with UDP-Gal. Based on the initial pretest of optimal buffers, MES buffer with 1% Triton X100 was selected to perform the binding affinity analysis. The measurements were performed at 22 °C in standard capillaries with 2% excitation power and 40% MST power. The initial fluorescence was uniform among the sixteen capillaries within a 10% tolerance threshold from the average (Fig. 2b). The thermographs showed no signs of aggregation or molecule adsorption to the capillary (Fig. 2c). The smooth binding curve relating the substrate concentration to the normalized fluorescence could be fitted based on a K_d_ model (Fig. 2d). Each point represents the mean of three sets of measurements. The binding of YFP-*At*GALS1 with UDP-Gal in MES buffer had an affinity of 112 ± 30 μM (± 68% confidence) (Fig. 2e), compared with data using Golgi-associated rat sialyltransferase ST-YFP [29], which served as a control protein and showed no significant binding with UDP-Gal. This data is in good agreement with the K_m_ of 142 ± 30 μM (± SE) measured by activity assay [28]. K_m_ and K_d_ are related as K_m_ = K_d_ + k_cat_/k_1_, based on standard Michaelis–Menten reactions, and often K_m_ is close to K_d_ because k_cat_ is low compared to k_-1_ [30].

### Analysis of substrates specificity of *At*GAL1

**Fig. 3:**
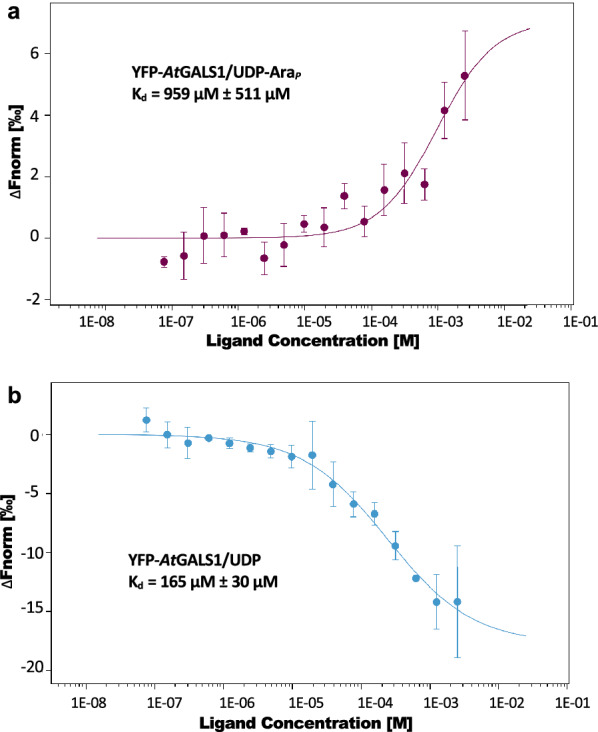
Substrate specificity assay of *At*GALS1. Dose–response curve for the binding interaction between YFP-*At*GALS1 and **a** UDP-Ara_*f*_, or **b** UDP. Concentration of YFP-*At*GALS1 was kept constant, while the ligand varied from 2.5 mM to 76.3 nM. Data were fitted to K_d_ model and confidence interval of the K_d_ is calculated from the variance of the fitted parameter, derived from Levenberg–Marquardt fit-algorithm. Error Bar: SD, n = 3. Values are the average ± 68% confidence

*At*GALS1 is reported as bifunctional, catalyzing the addition of galactose with UDP-Gal as substrate as well as termination of galactan chains by transferring arabinopyranose from UDP‐β‐l‐Ara_*p*_. However, UDP‐Gal is the preferred substrate with a tenfold lower Km [28]. To further screen the substrate specificity of *At*GALS1, we applied the optimized protocol to detect interaction of *At*GALS1 with nucleotide moiety and multiple nucleotide sugar donors. The interaction of *At*GALS1 with UDP‐Ara_*p*_ resulted in a calculated K_d_ of 0.96 ± 0.51 mM (± 68% confidence) (Fig. 3a), consistent with the K_m_ of 1.1 ± 0.2 mM previously reported [28]. Interestingly, UDP interacted with *At*GALS1 with a K_d_ of 165 ± 30 μM (± 68% confidence) (Fig. 3b). Since UDP is a product of the enzymatic reaction, it would appear that the reaction would produce a competitive inhibitor. However, in vivo the UDP concentration is kept low by the action of nucleoside diphosphatases [31]. No binding was detected with UDP-Ara_*f*_ or UDP-Xyl, indicating they cannot serve as substrates for *At*GALS1 (Additional file 1: Figure S2). These results underline the ability to screen the donor substrates of GTs in complex samples such as crude microsomes without purification.

### Acceptor binding preference of *At*GALS1

**Fig. 4:**
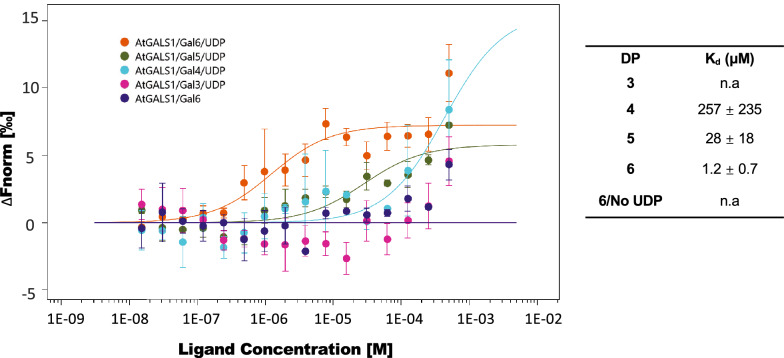
Acceptor binding assay of *At*GASL1 with β-1,4-galactooligosaccharides Gal_3-6_. *At*GALS1 expressed in *N. benthamiana* showed increasing binding affinity from DP4 to DP6 upon addition of UDP and no binding was detected without UDP. Gal_3_ did not bind

To test whether MST can also be used to detect acceptor binding of GTs, galactan substrates of different DP in the presence or absence of UDP were used for studying interaction with *At*GALS1. As evidenced in the results shown before (Fig. 3b), UDP can bind to the GT, and this would be expected to induce a conformational change that enables subsequent binding of the acceptor substrate [32]. The MST results showed that galactooligosaccharides longer than three residues can bind to *At*GALS1, and longer acceptors up to six residues have higher preferences, with higher binding affinities (Fig. 4). This result is consistent with the activity preference of *At*GALS1 for galactooligosaccharide substrates with a minimum of four galactose units and increasing activity with longer substrates (Gal_4‐6_) [28]. No binding of Gal_6_ could be detected in the absence of UDP.

### Substrate screening with selected *sorghum* GT61

**Fig. 5:**
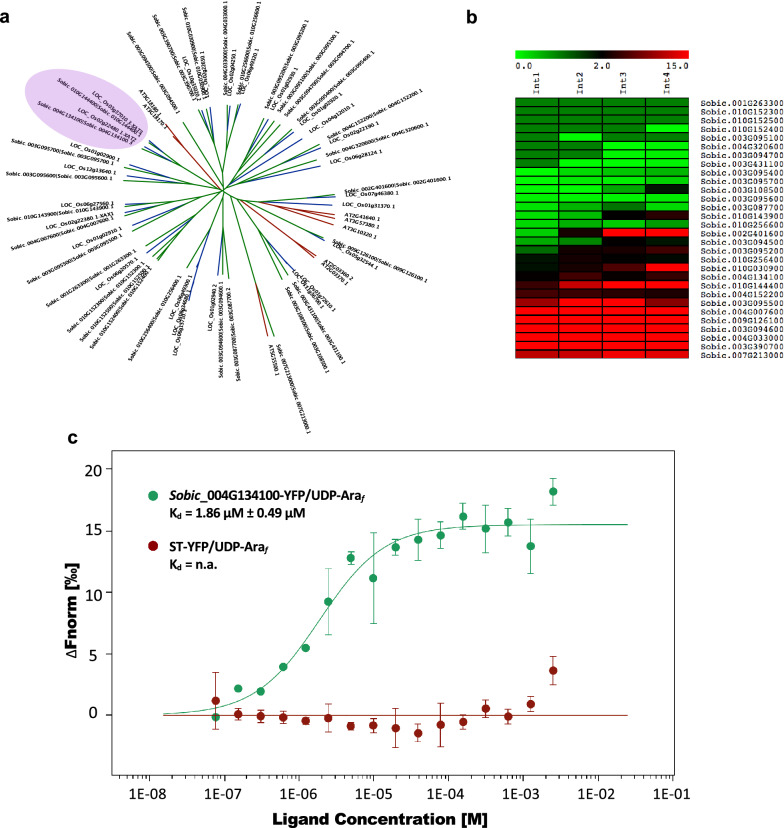
Substrate testing of sorghum GT61. **a** Combined phylogenetic tree of rice, *A. thaliana* and sorghum GT61 proteins. Purple circle highlights the clade containing *Os*XAT orthologs in sorghum. **b** Heatmap showing expression patterns of sorghum GT61 genes in growing internodes of sorghum. **c** Dose–response curve for the binding interaction between sorghum *Sobic*_004g134100 and UDP-Ara_*f*_. Error Bar: SD, n = 3. Values are the average ± 68% confidence

Sorghum (*Sorghum bicolor*) has emerged as a promising target for lignocellulosic biofuel production, but very little is known about the identities and functions of sorghum GTs. With a bioinformatics approach, we performed genome-wide identification of GT genes in sorghum based on the presence of the corresponding Pfam domains. The GT61 family is very large in grasses compared to dicots, and is known to include several members involved in decorating the xylan backbone [33, 34]. Since xylan is the predominant non-cellulosic polysaccharide in biomass, we were particularly interested in the GT61 family. To facilitate downstream biochemical and functional analysis, phylogenomic analysis was performed to prioritize candidates for in-depth characterization. In Fig. 5a, the phylogenetic clade highlighted in purple showed sorghum orthologs of rice *Os*XAT2 which have potential roles in arabinoxylan biosynthesis [34]. The expression patterns of these genes also suggested their roles in cell wall biosynthesis (Fig. 5b). Heterologous expression of *Os*XAT2 in *Arabidopsis* leads to arabinosylation of xylan, suggesting gain-of-function evidence for α-(1,3)-arabinosyltransferase activity [34]. However, direct biochemical evidence of the activity is still lacking. Here we selected one representative sorghum ortholog of *Os*XAT2, *Sobic*_004G134100, for substrate screening using MST. A binding curve of *Sobic*_004G134100-YFP with potential substrate UDP-Ara_*f*_ yielded a K_d_ of 1.86 μM ± 0.49 μM (± 68% confidence) while the control ST-YFP exhibited no detectable binding. The binding of UDP-Ara_*f*_ is much stronger than the binding of UDP-Gal to *At*GALS1 as would be expected since the cellular concentrations of UDP-Ara_*f*_ in leaves is about 30-fold lower than that of UDP-Gal [35]. *Arabidopsis* UDP-Ara_*f*_ transporters that transport UDP-Ara_*f*_ from the cytosolic compartment to the Golgi lumen have K_m_s in the 7-10 µM range [35]. UDP-Xyl, UDP-Ara_*p*_ and UDP-GlcA did not exhibit significant binding (Additional file 1: Figures S3 and S4). UDP sugar preparations often contain some free UDP and the very weak binding of UDP-GlcA (K_d_ > 0.1 M) could indicate binding of a small amount of free UDP in the sample rather than binding of UDP-GlcA. These results provide supporting evidence to predict the sorghum ortholog of *Os*XAT2 as an arabinofuranosyltransferase.

## Discussion

The identification and characterization of key enzymes associated with cell wall biosynthesis and modification is fundamental to gaining insights into cell wall dynamics. However, it is challenging that activity assays of GTs are very low throughput and acceptor substrates are generally not available. In this work we optimized and validated MST using the well-characterized enzyme *At*GASL1, and we also developed an analysis flow that uses MST-based strategies to screen potential substrates for novel GTs. We confirmed that in the presence of the appropriate nucleoside diphosphate, acceptor substrates can be screened in a similar way to donor substrates. In contrast to activity screens which must use two substrates, MST allows individual substrate screening to determine likely donors/acceptors of GTs. Furthermore, the detection of binding is a universal method, unlike activity assays which generally require different methods to detect activity depending on the product formed. Compared to other competitive techniques, such as saturation transfer difference nuclear magnetic resonance (NMR) [36], mass spectrometry (MS) [37] and isothermal titration calorimetry (ITC), MST is more straightforward, less time-consuming, and possesses high-throughput potential. A drawback is the relatively high cost of capillaries of about $12 per assay with 16 capillaries. Furthermore, in this assay, we expressed GTs as YFP-fusion proteins in *N. benthamiana* and optimized the MST method to allow the determination of substrate binding affinity without purification of the target protein from the cell lysate or heterologous expression system. This overcomes the obstacles of low quantities of native GTs in plants or inappropriate protein modification in a heterologous expression system, and at the same time causes no loss of enzymatic activity.

Thanks to the availability of both bioinformatics tools and full sequences of the sorghum genome, potential gene candidates that encode putative GTs for wall polysaccharide biosynthesis have been identified. As a proof of concept for the MST method we show that a sorghum GT from family GT61 can be identified as a likely arabinofuranosyltransferase, probably involved in xylan biosynthesis.

## Conclusions

In summary, our study optimized and validated MST to facilitate high throughput screening for GT substrates. A transient system in *N. benthamiana* was used which enables easy expression of fluorescently labeled protein. The filter combinations of the MST instrument determine which fluorophore is most suitable. The blue filter we used is suitable for GFP and YFP, and the green filter would be suitable for red fluorescing fluorophores. Expression in a plant system is an advantage since any necessary modification, e.g. *N*- and *O*-glycosylation, is likely to take place normally. The MST method was optimized to allow the determination of substrate binding affinity with crude microsomes, without purification of the target protein from the cell lysate. The buffer conditions we recommend should work well for Golgi-localized GTs. The pH of 6.5 of the recommended buffer is close to the pH of the Golgi in plants, and should also work for cytoplasm, ER, and plastid stroma, although a pH closer to 7 might be more optimal for those compartments [38]. We have only tested the method with several Golgi-localized GTs from plants, but we find it likely that good MST signal should be achievable for most GTs without further optimization. The MST method validated the interaction of UDP-Gal to the β-1,4-galactosyltransferase *At*GALS1, and showed consistent binding affinity as reported from activity assays. MST is also sensitive to capture the rare substrate of *At*GALS1 as UDP-Ara_*p*_ which is needed for termination of galactan and showed a tenfold lower affinity than UDP-Gal. The method of substrate screening we developed can be adapted to any plant species and is obviously not limited to GTs involved in cell wall biosynthesis. Obviously, binding of a nucleotide sugar does not prove that it is a substrate, but strong binding of non-substrates is unlikely to be common since that would cause non-productive competitive inhibition. Some GTs may not be entirely specific, as seen for example for *At*GALS1, which has low but biologically relevant activity with UDP-Ara_*p*_ and binds this substrate at relatively high but still physiologically meaningful concentrations. Therefore, binding data should be interpreted with caution, but a nucleotide sugar that does not bind at a physiologically relevant concentration is highly unlikely to be a substrate in vivo.

MST, as a rapid and sensitive method to screen the substrates of GTs, will greatly facilitate selection of candidates for further studies and engineering. The method will also be powerful in studying how interaction with other proteins, posttranslational modifications, allosteric regulators, or competitive inhibitors can affect the enzyme activity through altered substrate binding.

## Methods

### Chemicals

UDP-Gal and UDP-Xyl were of analytical grade and purchased from Sigma‐Aldrich. UDP‐β‐l‐Ara_*p*_ was purchased from Carbosource Service (Complex Carbohydrate Research Center, Athens, GA, USA) and and UDP‐β‐l‐Ara_*f*_ was purchased from Peptides International (Louisville, KY, USA). Galactan substrates Gal_n_ (n = 3–6) were chemically synthesized as previously described [39].

### Plasmid construction

cDNA of *Sobic*_004G134100 was amplified by PCR using primers as follows: F: 5′-CACCATGAAGGCGGTGGAG-3′ and R: 5′-TTGGTTCAATTGATCAAGAGCC-3′. A purified fragment was cloned into pENTR/D/TOPO entry vectors through GATEWAY LR reactions (Invitrogen), following the manufacturer’s protocol. *At*GALS1 and rat ST expression constructs were described previously [25, 40].

To generate transient protein expression constructs in *N. benthamiana*, the pENTR/D vector containing coding sequence of *Sobic*_004G134100 were recombined into pEarleyGate 101 [41] using LR Clonase II (Invitrogen). The binary vectors were electro-transformed into *Agrobacterium tumefaciens* strain GV3101::pMP90 for tobacco leaf infiltration.

### Transient expression in *Nicotiana benthamiana*

*Agrobacterium tumefaciens* strains harboring binary vectors were cultured in 10 ml LB medium overnight and harvested at 4500 rpm for 10 min. Concentrated cells were then washed by 10 ml of 10 mM MgCl_2_ twice and left at room temperature for 3 h prior to infiltration. Equal volumes of cell culture for expression of the gene of interest and cell culture expressing the p19 protein, which suppresses gene silencing [42] were mixed with an OD600 = 0.5 and infiltrated into the 4-week-old tobacco expanding leaves. The leaves were harvested 4 days after the infiltration for microsomal membrane preparation.

### Microsome extraction

Microsomal membranes were prepared according to the protocol previously described [43]. The *N. benthamiana* leaves transiently expressing *At*GASL1 or *Sobic*_004G134100 were ground in microsomal extraction buffer (50 mM HEPES‐KOH pH 7.0, 400 mM sucrose, 20 mM sodium ascorbate, 1 mM phenylmethylsulfonyl fluoride, 1% w/v polyvinylpolypyrrolidone). The Miracloth-filtered suspension was centrifugated at 3000×*g* for 10 min to remove cell debris. The supernatant was collected and centrifugated at 100,000×*g* for 1 h to isolate membranes. The precipitated pellet was then resuspended in buffer (50 mM HEPES‐KOH pH 7.0, 400 mM sucrose) and aliquots were flash‐frozen in liquid nitrogen and stored at − 80 °C until use. All above steps were performed at 4 °C. Total protein was measured by Coomassie Blue (Bio-Rad) assay based on bovine serum albumin standard.

### ANTS‐labeling and analysis of labeled galactan substrates

Each galactan oligosaccharide (200 μg) was reductively aminated with ANTS (Invitrogen) as follows: speedvac-dried oligosaccharides were resuspended in 5 μl of 0.2 M ANTS solution (in H_2_O:acetic acid = 17:3), 10 μl of dimethylsulfoxide (DMSO) buffer (30 μl acetic acid, 170 μl H2O, 200 μl DMSO) and 5 μl of 0.2 M 2‐picoline borane (in DMSO). After overnight incubation at 37 °C, samples were dried in a speedvac and resuspended in 100 μl H_2_O, as described in Mortimer et al. [44].

### PACE

Reactions were performed in a total of 25 μl containing MnCl_2_ (10 mm), Triton X‐100 (1% v/v) in buffer (50 mm MES, pH 6.5), 2 μg galactooligosaccharide substrate, 200 µM UDP‐Gal and microsomal membranes (50 μg total protein), as previously described [28]. Reactions were incubated at 30 °C for 2 h and then terminated by heating at 100 °C for 5 min, followed by centrifugation at 10,000×*g* for 10 min. Supernatants (15 µl) were mixed with 15 μl 3 m urea, and 5 μl samples were loaded on large format Tris‐borate acrylamide gel prepared as described previously [27], and electrophoresed at 200 V for 30 min followed by 1000 V for 1.5 h. The PACE gels were visualized with Syngene G‐box at Tumi-wavelength with a UV detection filter and long‐wave UV tubes (365 nm emission).

### MST buffer optimization

Various buffer solutions were tested to determine the best buffer composition based on stability and homogeneity within capillaries: HEPES buffer (50 mM HEPES pH 7.0; 5 mM MnCl_2_; 50 mM NaCl, 1% v/v Triton X-100), MES buffer (20 mM MES pH 6.5; 5 mM MnCl_2_, 1% v/v Triton X-100), MES buffer with extra KCl (20 mM MES pH 6.5; 5 mM MnCl_2_; 125 mM KCl, 1% v/v Triton X-100), MES buffer with Tween (20 mM MES pH 6.5; 5 mM MnCl_2_; 125 mM KCl, 0.05–2% v/v Tween 20) and PBS buffer (pH 7.4; 137 mM NaCl; 2.7 mM KCl; 10 mM Na_2_HPO_4_; 1.8 mM K_2_HPO_4_; 5 mM MnCl_2_; 0.05% Tween 20).

MST experiments were performed on a NanoTemper ^®^ Monolith NT.115 (NanoTemper Technologies, Germany) with blue/red filters. Samples were diluted 200X in different buffers listed above, and final concentration yield detectable fluorescent signals, between 200 and 1600 units of fluorescence (FI units). Prepared samples were loaded into standard treated capillaries for measurements using 40% MST power with laser off/on times of 0 s and 20 s, respectively, at 22 °C.

### Binding assay

To test the binding affinity between *At*GASL1 and UDP-Gal, microsomes containing YFP-*At*GASL1 were diluted in MES buffer (1% Tx100) to a final concentration of 1.5 mg/ml total protein. Diluted microsomes were centrifuged at 20,000×*g* for 10 min to remove potential protein aggregates. 10 µl of 5 mM UDP-Gal solution was diluted 1:1 in 10 µl buffer to make a 16-sample serial dilution from 2.5 mM to 76.3 nM. 10 µl of prepared microsome was then added to 10 µl of each ligand solution and incubated at room temperature for 10 min. For the acceptor binding between YFP-*At*GASL1 and Gal_3-6_, similar serial dilution of galactan substrates was prepared using a 1:1 dilution and the final concentration range from 0.5 mM to 15.3 nM, with the other parameters remaining the same. 2% excitation power and 40% MST power with laser off/on times of 0 s and 20 s were used in all MST experiments. All experiments were repeated three times for each measurement. Data analyses were performed using the MO.Affinity Analysis software (version 2.3, NanoTemper Technologies).

The binding constant K_d_ was calculated according to the protocol of NanoTemper Technologies. The fluorescence change in MST signal is normalized (Fnorm), defined as F_hot_/F_cold_ (F_hot_ as the hot region at 20 s after IR laser heating and F_cold_ as the cold region at 0 s). A dose–response curve is plotted as Fnorm against the ligand concentration. The K_d_ constants between a protein and its substrate was calculated using the saturation binding curve at equilibrium [45]. The fitting function is derived from the law of mass action:

1 $$f\left( {Concentration} \right) = Unbound + \frac{{\left( {Bound - Unbound} \right)(\left[ {L_{tot} } \right] + \left[ {P_{tot} } \right] + K_{d} - \sqrt {\left( {\left[ {L_{\text{tot}} } \right] + \left[ {P_{\text{tot}} } \right] + \left[ {K_{d} } \right]} \right)^{2} - 4\left[ {L_{\text{tot}} } \right].\left[ {P_{\text{tot}} } \right]} }}{{2\left[ {P_{\text{tot}} } \right]}}$$

unbound is the response value of unbound state; bound is the response value of bound state; [P_tot_] is the total protein concentration; [L_tot_] is the total ligand concentration; K_d_ is the dissociation constant

### Sequence retrieval and phylogenetic analysis

The Hidden Markov Model (HMM) profile for GT61 proteins (PF04577) was downloaded from Pfam Database (http://pfam.xfam.org/) and used as a query to search against sorghum proteome available at Phytozome (http://www.phytozome.net/) using HMMER V3.1b1 (http://hmmer.org/) with default parameters. The information about GT61 proteins of rice and *Arabidopsis* was obtained from the previously published studies [33, 46]. Corresponding rice and *Arabidopsis* protein sequences were retrieved from rice genome annotation project database (http://rice.plantbiology.msu.edu/) and The *Arabidopsis* Information Resource (TAIR) database (https://www.arabidopsis.org/), respectively. GT61 proteins of sorghum, rice and *Arabidopsis* were aligned using ClustalX [47] and a neighbor-joining phylogenetic tree was generated using default parameters.

### Expression analysis

For expression analysis of GT61 genes, we used publicly available RNA Seq-based expression data corresponding to four internodes of Sorghum [48]. The normalized expression was downloaded from NCBI-GEO (accession number GSE98817) and expression values for GT61 genes in sorghum was extracted. The expression heatmap was generated using MeV microarray data analysis platform (http://www.tm4.org/mev/).

## Supplementary information

**Additional file 1: Figure S1.** Thermographs corresponding to the different buffer conditions shown in Table 1. **Figure S2.***At*GALS1 expressed in *Nicotiana benthamiana* has no detectable binding of UDP-Ara_*f*_. **Figure S3.***Sobic_*004G134100 *At*GALS1 has no detectable binding of UDP-Ara_*p*_ or UDP-Xyl. **Figure S4.***At*GALS1 has very weak interaction with UDP-GlcA at physiologically irrelevant concentrations.

## Data Availability

All data generated or analyzed during this study are included in this published article and its additional files, or are available from the corresponding authors per request. All constructs are available via the JBEI registry (https://registry.jbei.org/).

## Abbreviations

GT: Glycosyltransferase
MST: Microscale thermophoresis
DP: Degree of polymerization
ANTS: 8-Amino-naphthalene-1,3,6-trisulfonic acid
