# Supplementary material for: Microscale thermophoresis as a powerful tool for screening glycosyltransferases involved in cell wall biosynthesis
Source: Plant Methods. 2020 Jul 28;16:99. doi: 10.1186/s13007-020-00641-1 (PMC7389378; doi:10.1186/s13007-020-00641-1)
Supplement: Supplementary file 1 — Additional file 1: Figure S1. Thermographs corresponding to the different buffer conditions shown in Table 1. Figure S2.AtGALS1 expressed in Nicotiana benthamiana has no detectable binding of UDP-Araf. Figure S3.Sobic_004G134100 AtGALS1 has no detectable binding of UDP-Arap or UDP-Xyl. Figure S4.AtGALS1 has very weak interaction with UDP-GlcA at physiologically irrelevant concentrations. [file 13007_2020_641_MOESM1_ESM.pdf]

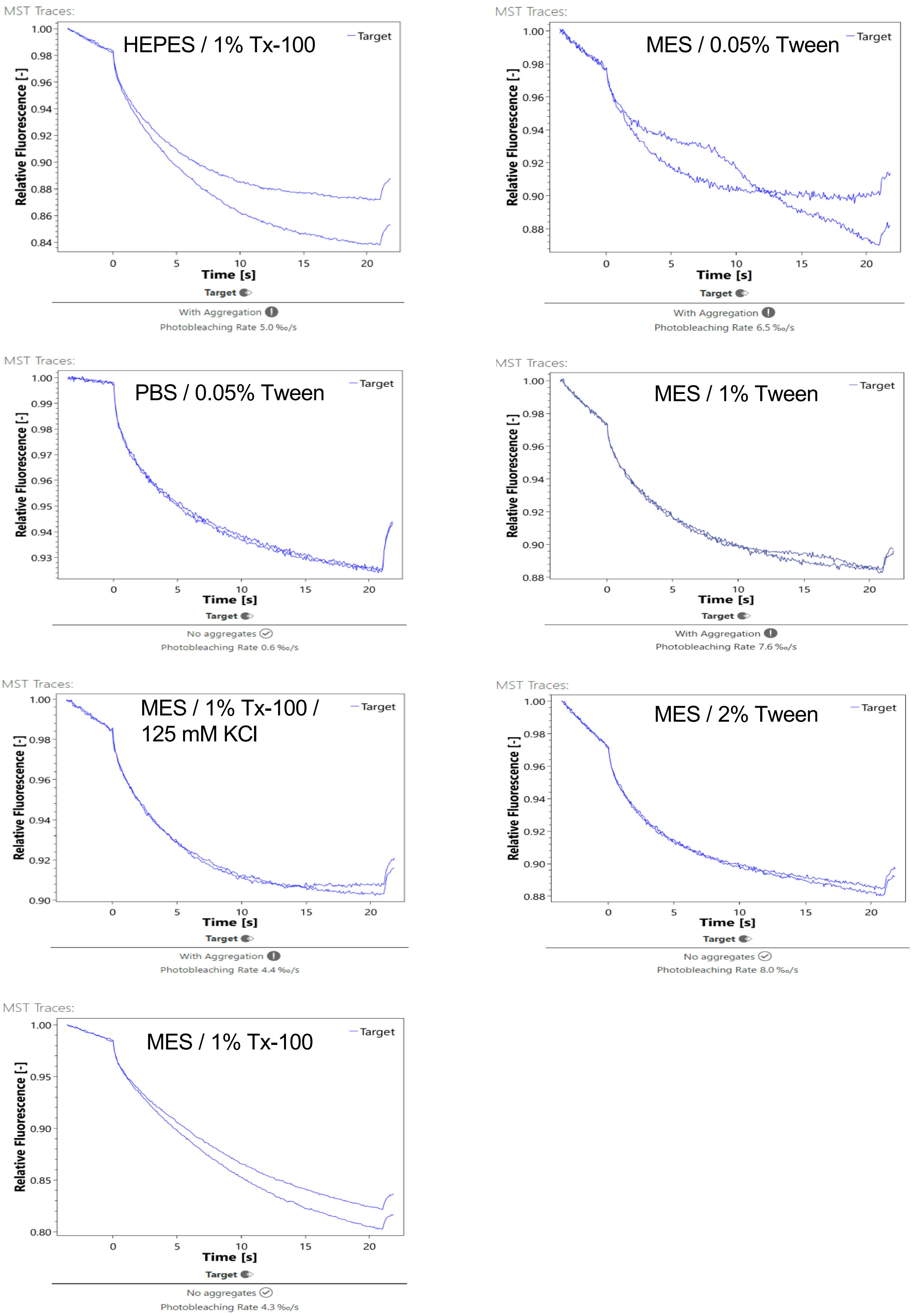

Fig S1. Thermographs corresponding to the different buffer conditions shown in Table 1. Some of the conditions resulted in bumpy curves indicating aggregation. MES / 1% Triton X-100 did not show aggregation and had higher fluorescence yield. Therefore, this condition was chosen for subsequent experiments

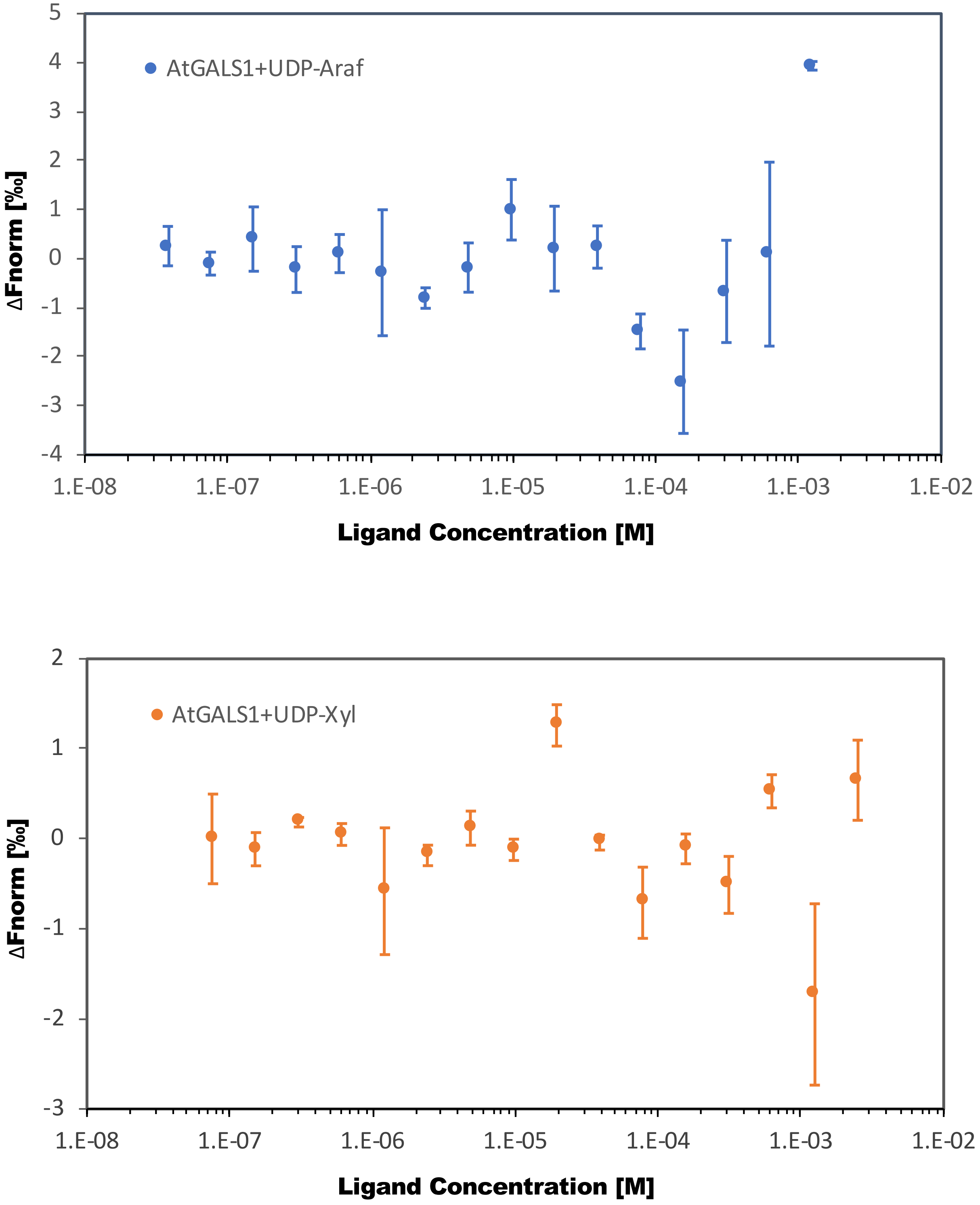

Fig S2. *At*GALS1 expressed in *Nicotiana benthamiana* has no detectable binding with UDP-Ara$_f$ or UDP-Xyl.

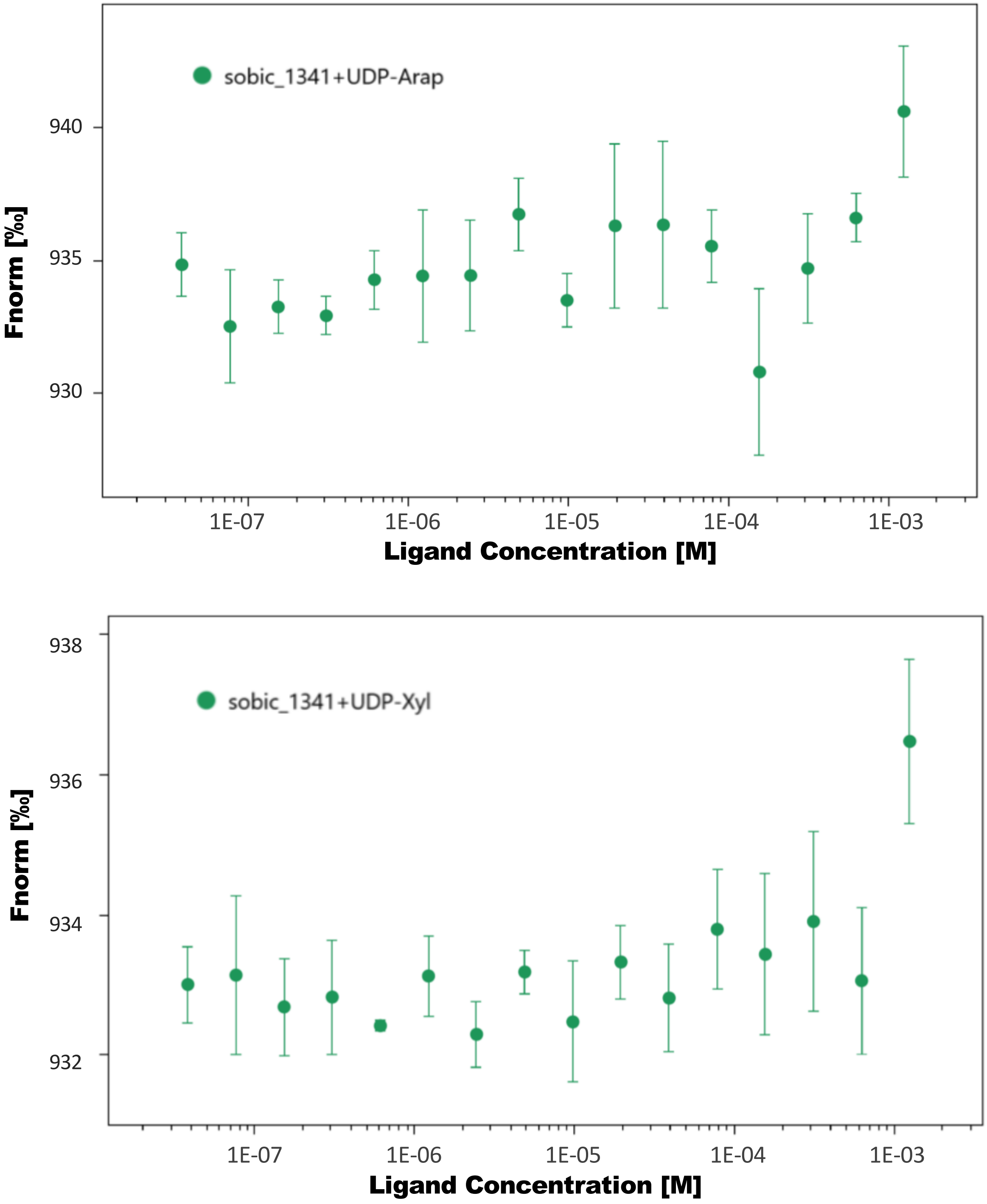

Fig S3. *Sobic*_004G134100 expressed in *Nicotiana benthamiana* has no detectable binding with UDP-Ara$_p$ or UDP-Xyl.

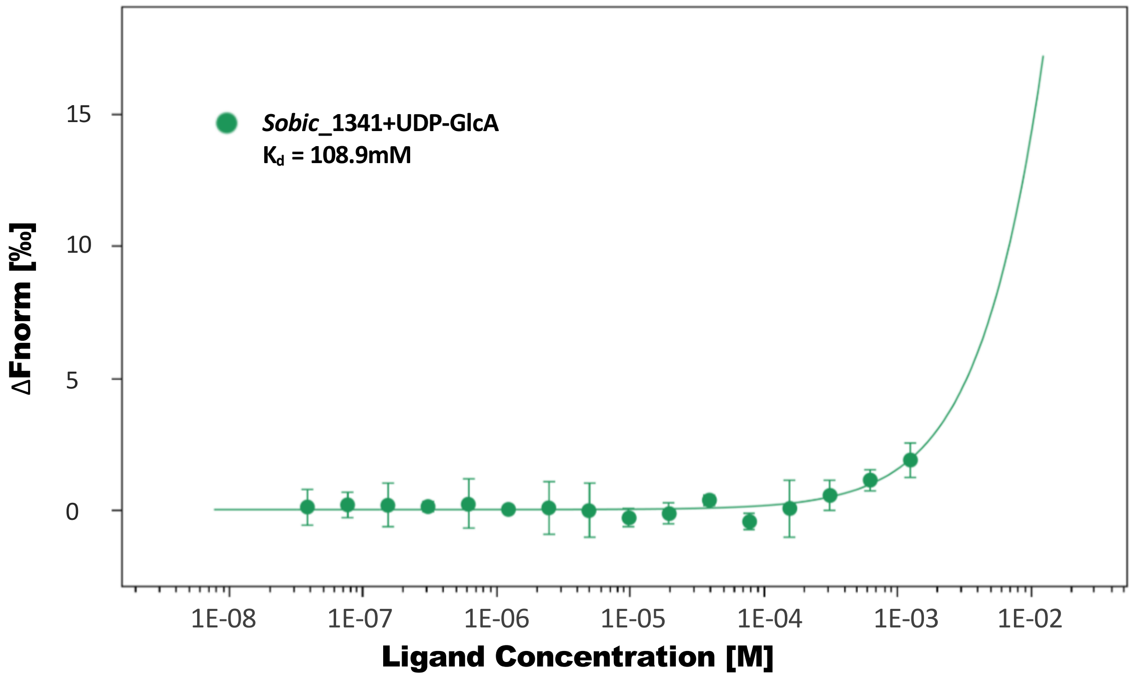

Fig S4. *Sobic*_004G134100 expressed in *Nicotiana benthamiana* has very weak binding with UDP-GlcA (K_d > 0.1 M).
